# Commentary: Probiotic and technological properties of *Lactobacillus* spp. strains from the human stomach in the search for potential candidates against gastric microbial dysbiosis

**DOI:** 10.3389/fmicb.2015.00433

**Published:** 2015-05-19

**Authors:** Amit K. Tyagi, Sahdeo Prasad

**Affiliations:** Cytokine Research Laboratory, Department of Experimental Therapeutics, The University of Texas MD Anderson Cancer Center Houston, TX, USA

**Keywords:** *Lactobacillus reuteri*, *Helicobacter pylori*, probiotics, inflammation, triple therapy

*Helicobacter pylori*, a curved-shaped, flagellated, microaerophilic, gram-negative bacillus is naturally colonized bacteria in humans. This bacterium can be found in 25–50% of the population in developed countries and in 70–90% in developing countries, probably, due to the poor hygiene (Go, 2002). New epidemiological studies revealed that the prevalence of *H. pylori* is declining. However, *H. pylori*-infected population develops various diseases including peptic ulcer, chronic gastritis, and gastric mucosa-associated lymphoid tissue (MALT) lymphoma (Ruggiero, 2014). It is the only bacterium that has been linked to the gastric cancer and ulcer disease, and thus associated with significant morbidity and mortality worldwide. Moreover, till now there is no universally effective therapeutic regimen and vaccine are available for the treatment of *H. pylori* associated diseases. Although triple therapy (combining acid suppression with clarithromycin, amoxicillin, or nitroimidazolic compounds) and antibiotics are being used against *H. pylori* infection, prolongation of these therapeutic agents is declining worldwide because of their side effects (such as vomiting, diarrhea, nausea, constipation, headache, etc.) (Yuan et al., 2013) and prevalence of antimicrobial resistance. *H. pylori* eradication treatments are still remain a challenge.

Extensive research on *H. pylori* and associated diseases concluded that probiotics can be used as an alternative or complementary therapy for the management of *H. pylori* infection (Emara et al., 2014), since it does not cause side effects as triple therapy. Probiotics have potential to maintain the disturbed gastroenterological conditions and also beneficial for the patients of inflammatory bowel disease, ulcerative colitis, Crohn's disease, antibiotic-, and rotavirus-associated diarrhea, adenocarcinoma, colorectal cancer, etc. (Goossens et al., 2003). An improvement of *H. pylori* associated gastric inflammation by the use of probiotics could be contributed by various mechanisms (Emara et al., 2014). Accumulated evidences suggested that *H. pylori* could be eradicated from the stomach by selective bacterial–bacterial cell interaction. *Lactobacillus reuteri* was identified as a highly specific binding antagonist to *H. pylori* among *Lactobacillus* species. *L. reuteri* DSM17648 strain co-aggregates with different *H. pylori* strains and serotypes. However, it does not affect other intestinal and commensal oral bacteria (Holz et al., 2015).

How *L*. *reuteri* acts against *H. pylori* is not completely uncovered. However, it is reported that *L*. *reuteri* produces a compound called reuterin, which work as an antimicrobial agent. Other than reuterin, *L. reuteri* also produces some potent antimicrobial compounds, such as reutericin 6 and reutericyclin, but these have no effects on Gram-negative bacteria (Ganzle, 2004). *L. reuteri* also inhibits the binding of *H. pylori* to the putative glycolipid receptors. Mukai et al. (2002) examined the binding competition of *L. reuteri* strains, (JCM1081 and TM105) and *H. pylori* to gangliotetraosylceramide (asialo-GM1) and sulfatide. It was identified as a possible sulfatide-binding protein of the *L. reuteri* strain.

*L. reuteri* helps to improve the eradication rate of *H. pylori* induced by triple therapy (Table 1). In a recent clinical trial, it was observed that triple therapy supplemented with *L. reuteri* increased the eradication rate of *H. pylori* by 8.6%, as well as reduced the side effects and improve the GSRS (Gastrointestinal Symptom Rating Scale) score (Emara et al., 2014). A new probiotic having two strains of *L. reuteri* (DSM 17938 and ATCC PTA 6475) were also studied for the inhibitory effect of *H. pylori* growth, gastritis and prevention of antibiotic-associated side effects, when administered with triple therapy. *L. reuteri* combination increased the eradication rate by 9.1% (Francavilla et al., 2014). Sequential therapy with *L. reuteri* supplementation was also reported in the eradication treatment of *H. pylori* and the intensity of antibiotic-associated side effects (Efrati et al., 2012). *H. pylori* infection was also defined as positive gastric histopathology ^13^C urea breath test (^13^C-UBT). Dore et al. (2014) have done an open label single center study, where they found a significant reduction in urease activity with a difference of mean of 38.8 vs. 25.4 assessed before and 4–6 weeks post therapy. In another study, *L. reuteri* DSMZ17648 was also reported to co-aggregates with *H. pylori in vitro* and was shown to reduce ^13^C-UBT *in vivo* (Mehling and Busjahn, 2013).

**Table 1:** Selected clinical trials using *Lactobacillus reuteri* for *H. pylori* eradication treatment.

**Treatment**	**Probiotic(s)**	**Eradication rate**	**Probiotic efficacy**	**References**
Triple therapy, Omeprazole 20 mg, amoxicillin 1 g, clarithromycin 500 mg, 14 d	*L. reuteri* ATCC PTA 6475, *L. reuteri* DSM 17938, 14 d during therapy + further 14 d, Control	74.3% (26/35) 65.7% (23/35)	Non-significant increase of eradication rate with improved GSRS score and reduction of side effects (taste disorder, diarrhea)	Emara et al., 2014
Three-phase study; pre-eradication (1–28 d), eradication (29–35 d), follow-up (36–96 d), Triple therapy	*L. reuteri* ATCC PTA 6475, *L. reuteri* DSM 17938, Control	75% (37/50) 65.9% (33/50)	Non-significant increase of eradication rate but no difference in GSRS score	Francavilla et al., 2014
Pantoprazole 20 mg, 8 weeks	*L. reuteri*, 8 weeks	14.2% (3/21)	Good tolerability with no side effects	Dore et al., 2014
Levofloxacin 500 mg, esomeprazole 20 mg, amoxicillin 1 g, 7 d	*L. reuteri*, during therapy + further 7 d Control	80% (36/45) 62.2% (28/45)	Significantly increase of eradication rates and reduction of side effects (Nausea, diarrhea)	Ojetti et al., 2012
Omeprazole 1 mg/kg, amoxicillin 50 mg/kg, clarithromycin15 mg/kg, 7 d	*L. plantarum, L. reuteri, L. casei* subsp*. rhamnosus, B. infantis, and B. longum, L. acidophilus, L. salivarius, S. thermophilus, L. sporogenes*, during therapy Control	82.2% (30/34) 76.4% (26/34)	Non-significant increase of eradication rates; significant reduction of side effects (epigastric pain, nausea, vomiting, diarrhea)	Tolone et al., 2012
Pantoprazole 20 mg, amoxicillin 1 g, clarithromycin 500 mg, Triple therapy, 7 d Sequential regimen, 10 d	*L. reuteri* ATCC55730, during therapy + further 7 or 10 d	63% (52/83) 88% (73/83)	Significantly higher eradication rate and reduction of side effects in sequential regimen	Efrati et al., 2012
Sequential therapy (Details not describe)	*L. reuteri* ATCC55730, 8 weeks Control	33.8 ± 15% (33) 35.8 ± 15.5% (33)	Significant decrease in Gastrointestinal Symptom	Francavilla et al., 2008
Triple therapy (Details not describe)	*L. reuteri*, 7 d Control	63% 53%	Lowest incidence of side-effects	Scaccianoce et al., 2008
No drug	*L. reuteri* SD2112, 8 weeks	69.7 ± 4% (33)	Significant reduction of ^13^C-UBT	Imase et al., 2007
Omeprazole 1 mg/kg, amoxicillin 50 mg/kg, clarithromycin15 mg/kg, sequential therapy, 10 d	*L. reuteri* ATCC55730 (SD2112) Control	85% (17/20) 80% (16/20)	Significant reduction of GSRS score	Lionetti et al., 2006

*GSRS, Gastrointestinal Symptom Rating Scale; ^13^C-UBT; ^13^C Urea Breath Test*.

Antibiotic-associated gastrointestinal side effects are major drawbacks of all *H. pylori* therapies. One of them is levofloxacin-based second-line therapy. The efficacy of *L. reuteri* supplementation during a second-line levofloxacin triple therapy for *H. pylori* eradication was reported by Ojetti et al. (2012). In this study, The *H. pylori* eradication rate was significantly increased (18%) and incidence of nausea and diarrhea was significantly lowered due to the *L. reuteri* supplementation. The therapeutic role of *L. reuteri* was also compared with a high concentration of probiotics for *H. pylori* eradication, where the incidence of side effects was lowest in 7-day therapy plus *L. reuteri* (6%) treated group (Scaccianoce et al., 2008). These studies show that *L. reuteri* can be used as a complementary therapy for *H. pylori* infection. *L. reuteri* may compete directly with *H. pylori*, possibly by interference with adherence or by the production of antimicrobial molecules.

In a recent “Frontiers in Microbiology” paper by Delgado et al. (2014), authors isolated 10 strains of different *Lactobacillus* species from the gastric biopsies and stomach juice samples of healthy humans. These all strains were tested for their functional properties, like bile tolerance, acid resistance, adhesion to epithelial gastric cells, production of antimicrobial compounds, antioxidative activity, antibiotic resistance, carbohydrate fermentation, glycosidic activities, and inhibition of *H. pylori*. *In vitro*, two gastric strains (particularly LR32 and LR34) showed good survival under gastrointestinal conditions, along with strong anti-Helicobacter and antioxidative activities. Thus, these strains can be considered as promising probiotic candidates.

The data presented have important implications for different disciplines including gastroenterology, microbial ecology, colorectal cancer, nutrition and health. Thus, based on these findings following intriguing questions can be raised: (1) To what extent *L. reuteri* is helpful for the prevention of *H. pylori*-induced colorectal cancer? (2) If it is available in our stomach or gut, how *L. reuteri* population can be increased inside the gut? (3) What could be the other potential probiotics and nutraceuticals, which can synergize the growth of *L. reuteri* in the gut?

## Conflict of interest statement

The authors declare that the research was conducted in the absence of any commercial or financial relationships that could be construed as a potential conflict of interest.
