# Corrigendum: Commentary: Probiotic and technological properties of *Lactobacillus* spp. strains from the human stomach in the search for potential candidates against gastric microbial dysbiosis

**DOI:** 10.3389/fmicb.2019.01628

**Published:** 2019-07-18

**Authors:** Amit K. Tyagi, Sahdeo Prasad

**Affiliations:** Cytokine Research Laboratory, Department of Experimental Therapeutics, The University of Texas MD Anderson Cancer Center, Houston, TX, United States

**Keywords:** *Lactobacillus reuteri*, *Helicobacter pylori*, probiotics, inflammation, triple therapy

In the original article, there was error in Table 1 as published. The probiotic efficacy for Emara 2014 and Francavilla 2014 reported a significant increase instead of a non-significant increase.

**Table 1:** Selected clinical trials using *Lactobacillus reuteri* for *H. pylori* eradication treatment.

**Treatment**	**Probiotic(s)**	**Eradication rate**	**Probiotic efficacy**	**References**
Triple therapy, Omeprazole 20 mg, amoxicillin 1 g, clarithromycin 500 mg, 14 d	*L. reuteri* ATCC PTA 6475, *L. reuteri* DSM 17938, 14 d during therapy + further 14 d, Control	74.3% (26/35) 65.7% (23/35)	Non-significant increase of eradication rate with improved GSRS score and reduction of side effects (taste disorder, diarrhea)	Emara et al., 2014
Three-phase study; pre-eradication (1–28 d), eradication (29–35 d), follow-up (36–96 d), Triple therapy	*L. reuteri* ATCC PTA 6475, *L. reuteri* DSM 17938, Control	75% (37/50) 65.9% (33/50)	Non-significant increase of eradication rate but no difference in GSRS score	Francavilla et al., 2014
Pantoprazole 20 mg, 8 weeks	*L. reuteri*, 8 weeks	14.2% (3/21)	Good tolerability with no side effects	Dore et al., 2014
Levofloxacin 500 mg, esomeprazole 20 mg, amoxicillin 1 g, 7 d	*L. reuteri*, during therapy + further 7 d Control	80% (36/45) 62.2% (28/45)	Significantly increase of eradication rates and reduction of side effects (Nausea, diarrhea)	Ojetti et al., 2012
Omeprazole 1 mg/kg, amoxicillin 50 mg/kg, clarithromycin15 mg/kg, 7 d	*L. plantarum, L. reuteri, L. casei* subsp*. rhamnosus, B. infantis, and B. longum, L. acidophilus, L. salivarius, S. thermophilus, L. sporogenes*, during therapy Control	82.2% (30/34) 76.4% (26/34)	Non-significant increase of eradication rates; significant reduction of side effects (epigastric pain, nausea, vomiting, diarrhea)	Tolone et al., 2012
Pantoprazole 20 mg, amoxicillin 1 g, clarithromycin 500 mg, Triple therapy, 7 d Sequential regimen, 10 d	*L. reuteri* ATCC55730, during therapy + further 7 or 10 d	63% (52/83) 88% (73/83)	Significantly higher eradication rate and reduction of side effects in sequential regimen	Efrati et al., 2012
Sequential therapy (Details not describe)	*L. reuteri* ATCC55730, 8 weeks Control	33.8 ± 15% (33) 35.8 ± 15.5% (33)	Significant decrease in Gastrointestinal Symptom	Francavilla et al., 2008
Triple therapy (Details not describe)	*L. reuteri*, 7 d Control	63% 53%	Lowest incidence of side-effects	Scaccianoce et al., 2008
No drug	*L. reuteri* SD2112, 8 weeks	69.7 ± 4% (33)	Significant reduction of ^13^C-UBT	Imase et al., 2007
Omeprazole 1 mg/kg, amoxicillin 50 mg/kg, clarithromycin15 mg/kg, sequential therapy, 10 d	*L. reuteri* ATCC55730 (SD2112) Control	85% (17/20) 80% (16/20)	Significant reduction of GSRS score	Lionetti et al., 2006

*GSRS, Gastrointestinal Symptom Rating Scale; ^13^C-UBT; ^13^C Urea Breath Test*.

Additionally, the reference for “Francavilla et al., 2008” in Table 1 was incorrectly written as “Francavilla, R., Lionetti, E., and Cavallo, L. (2008). Sequential treatment for Helicobacter pylori eradication in children. *Gut* 57:1178.” It should be “Francavilla, R., Lionetti, E., Castellaneta, S. P., Magistà, A. M., Maurogiovanni, G., Bucci, N., et al. (2008). Inhibition of *Helicobacter pylori* infection in humans by *Lactobacillus* reuteri ATCC 55730 and effect on eradication therapy: a pilot study. *Helicobacter* 13, 127–134. doi: 10.1111/j.1523-5378.2008.00593.x”.

The corrected Table 1 and Reference appear below.

The authors apologize for this error and state that this does not change the scientific conclusions of the article in any way. The original article has been updated.
